# Certificate-Of-Need Regulation and Healthcare Service Quality: Evidence from the Nursing Home Industry

**DOI:** 10.3390/healthcare8040423

**Published:** 2020-10-23

**Authors:** Bichaka Fayissa, Saleh Alsaif, Fady Mansour, Tesa E. Leonce, Franklin G. Mixon

**Affiliations:** 1 Department of Economics and Finance, Middle Tennessee State University, Murfreesboro, TN 37132, USA; bichaka.fayissa@mtsu.edu; 2 Department of Economics, University of Hail, Hail 50141, Saudi Arabia; SS.alsaif@uoh.edu.sa; 3 Center for Economic Education, Columbus State University, 4225 University Avenue, Columbus, GA 31907, USA; mansour_fady@columbusstate.edu (F.M.); leonce_tesa@columbusstate.edu (T.E.L.)

**Keywords:** certificate-of-need regulation, service quality, theory of economic regulation, nursing homes, health economics

## Abstract

This quantitative study investigates the effect of certificate-of-need (CON) regulation on the quality of care in the nursing home industry. It uses county-level demographic data from the 48 contiguous US states that are extracted from the American Community Survey (ACS) and cover the years 2012, 2013, and 2014. In doing so, it employs a new set of service quality variables captured from a variety of county-level data sources. Instrumental variables results indicate that health survey scores for nursing homes that are computed by healthcare professionals are about 18–24% lower, depending on the type of nursing home under consideration, in states with CON regulation. We also find that the presence of CON regulation leads to a substitution of lower-quality certified nursing assistant care for higher-quality licensed practical nurse care, regardless of the type of nursing home under consideration.

## 1. Introduction

In the past 25 years, the increasing cost of nursing home care has been a major concern for municipalities and state legislatures in the US. Nursing homes have not only contributed to the growing Medicaid expenditures of post-acute care, the cost of long-term care has continued to grow overwhelmingly, capturing an enormous amount of Medicaid expenditures. The share of total Medicare spending on skilled nursing facility care increased from 1 to 3%, while the share of total Medicare spending on home health care increased from 2 to 3%. Additionally, between 1994 and 2009, average spending on post-acute care more than doubled for most diagnoses [1]. Yet, despite these increases, the nursing home industry continues to be considered as alternative housing for seniors and a long-term care option for older Americans [2]. As such, legislatures are faced with escalating pressure to increase the capacity of nursing homes and other health services [3,4]. Lastly, economic theory suggests that there is a positive correlation between quality and demand in the presence of high price elasticity of demand, as in the case of nursing homes. According to Chiswick [5], the price elasticity of demand for nursing homes is at −2.2, which is relatively high.

Concerns related to overcharging patients or convincing elderly citizens to commit to being institutionalized unnecessarily in order to cover the expenses in a saturated competitive nursing home market compelled many states to implement certificate-of-need (CON) regulations in the late 1980s. The regulations allow for enough nursing homes to meet the actual capacity of the market or the necessary demand, based on their targeted population (i.e., the population over the age of 65 years old. The CON regulations were enacted to control nursing home costs through various forms of entry barriers. However, research by Nyman [6] finds that CON regulations are associated with more Medicaid violations, while Rahman, Galarraga, Zinn, Grabowski, and Mor [7] find that both Medicaid and Medicare spending have increased in the CON states. This study investigates the relationship between CON regulation and the quality of service in the nursing home industry. In this regard, our study follows a stream of research on the impact of CON regulations on the dialysis industry [8,9,10,11]. Our analysis focuses on for-profit nursing homes as they represent the dominant long-term care type ownership in the industry. Furthermore, for-profit nursing homes are expected to demonstrate a relatively high level of efficiency, including reduced employment of more expensive labor hours, in order to control cost in a less competitive market. This study hypothesizes that the various entry barriers associated with CON regulation reduce competition among nursing homes, which in turn puts downward pressure on the quality of service provided. Using a variety of outcome variables to measure the quality of the service provided by nursing homes, this study tests the hypothesis that nursing homes in CON states tend to be less adapted to meet the quality-of-service outcomes exhibited by nursing homes in other states.

Instrumental variables results discussed below indicate that health survey scores for nursing homes that are computed by healthcare professionals are lower in states with CON regulation, depending on the type of nursing home. We also find that the presence of CON regulation leads to a substitution of lower-quality certified nursing assistant care for higher-quality licensed practical nurse care, regardless of the type of nursing home under consideration. Before turning to our results, we first provide a brief review of prior literature concerning economic regulation of the nursing home industry.

## 2. Literature Review: A Brief Review

In addition to various federal policy changes, many states have reacted to the increasing cost of long-term nursing home care, along with other changes in the market for nursing home care, by regulating the number of nursing homes and nursing home services [12,13]. The most significant regulation has been the certificate-of-need (CON) program, which requires the approval of state regulatory officials for either the establishment of a new nursing home facility or the growth of an existing nursing home service provider, whether that is physical structural expansion or an increase in the scope of services. The idea of enforcing a supply control is founded on Roemer’s Law, following the belief that utilization and supply are positively correlated, meaning that when utilization rises, supply increases, regardless of the actual population’s need [14,15]. The quality of service provided by nursing homes remains a concern even in an environment of changing regulations.

The most commonly accepted yardstick for measuring health care quality was established by Donabedian [16], in which three different classifications of quality standards were defined: the structure of the medical facility, the process of services, and the clinical outcomes. This outline was initially established for the analysis of medical care service and has been broadly used in studies on nursing homes. Structural assessment explores the characteristics of the location in which the care is being provided. The structure of the medical facility incorporates the physical features of the facility, classifications of the nurses, and the managerial staffing at a given facility. The process of the services includes the nature and magnitude of services offered to patients in compliance with the accepted principles of proper care for particular conditions. According to Donabedian [16], the interdependency between the three quality measurements is unequivocal—that is, a good structure increases the likelihood of proper process and a suitable process enhances the probability of a good outcome [16].

Staff-to-resident ratios and costs are two variables that have been used as predictors of service quality. Higher staff-to-resident ratios are found in some studies to be indicative of higher service quality [17,18] and are used as predictors of lower mortality rates [19]. Likewise, expenditures on nursing home care are used as indicators of quality (i.e., high expenditures imply premium quality). As a result, no consensus exists on a comprehensive set of the measures of quality that can be used as a substitute for the structure or to process measures of care provided by nursing homes [20].

A variety of outcome variables have been used to measure the quality of services provided by nursing homes. Gertler [21,22], for example, uses three input measures of quality: hundreds of hours of nursing labor, hundreds of hours of other labor, and a supply quantity index. Both types of working hours are adjusted for efficiency variations across nursing homes in order to address any concern that more labor hours may not necessarily mean that a nursing home’s services are of higher quality. In some cases, more labor may simply mean that a nursing home is less efficient. Using the 1987 Institutional Population Component of the National Medical Expenditure Survey, Cohen and Spector [23] estimate the effect of Medicaid reimbursement rates on staff intensity, as well as the effect of staff intensity on resident outcomes, in order to investigate whether the intensity of staff working hours results in better outcomes. Quality is distinguished in this study using three proxies—registered nurses (RNs) per 100 residents, licensed practical nurses (LPNs) per 100 residents, and total nursing staff per 100 residents. Three outcome measures are then estimated—mortality rate, the presence of a pressure sore, and a change in functional status. Lastly, some studies use process measures, such as the rates of drug inaccuracy incidents, the level of physical mobility limitations, the usage of catheters and feeding tubes, and the number of nursing home deficiency citations [24,25].

There is also no consensus in previous research as to the impact of tighter regulations on the quality of service provided by nursing homes. Zinn [26] examined the impact of competition on nursing home quality and found that statewide regulation results in a lower level of quality of care [26]. Similarly, using data from the 1987 Medicare and Medicaid Automated Certification Survey (MMAC), Aaronson, Zinn, and Rosko [27] found that restrictions on nursing home construction lead to lower staffing of registered nurses and a higher percentage of residents who are physically underserved. This study controlled the variation in market concentration, demographics, and Medicaid policies, as well as resident and facility characteristics. Using 1983 data from Wisconsin, Nyman [28] found that tighter bed supply regulation is associated with a higher rate of Medicaid violations in nursing homes.

Finally, contrary to the previous findings, evidence presented by Gertler [21,22] that controls for residents, facilities, economic conditions, demographics, and market characteristics suggests that Medicaid reimbursement rates improve access for Medicaid residents. There is some indication in Gertler’s results [21,22] that the increased access is to a lower quality service. Gertler and Waldman [29] found similar results using total Medicaid expenditures as a measure of nursing home service quality. Using 1995–1996 data on all US Medicaid-certified nursing homes, Grabowski [24,25] showed that an increase in Medicaid reimbursement rates leads to a modest increase in nursing home service quality.

In terms of more recent research, survey evidence analyzed by Ko and Chou [30] revealed noteworthy shortcomings in nursing home service quality. Respondents indicated that both the physical condition and the temperature in the residents’ rooms were unsuitable and that the medical treatments and doctor visits were not well scheduled. The residents in the Taiwan-based nursing homes also reported that the staff did not solve their problems sincerely or clearly understand their needs [30]. Relatedly, De Boer et al. [31] collected survey evidence from Japan in order to examine the relationship between the physical environment and the well-being of nursing home residents with dementia. In doing so, they compared the physical environments of different types of nursing homes, including traditional nursing homes, small-scale living facilities, and green care farms. Interestingly, while they found that physical environment is important to the quality of care in nursing homes, with particular attention to small-scale living facilities, having a potentially beneficial physical environment does not necessarily lead to optimal use of the environment, as some areas of a nursing home (e.g., outdoor areas) are not fully utilized [31].

A recent study by Unroe, Stump, Effler, et al. [32] compared service quality differences between nursing homes and other types of facilities, including at-home care. More specifically, they examined differences in perceived quality of hospice care for individuals living at home or in a nursing home or an assisted living facility. Using hospice medical record data and survey data from Family Evaluation of Hospice Care for more than 7500 individuals, they found that hospice care provision in nursing homes was generally viewed as inferior to that in assisted living facilities [32]. Finally, a new study by Bowblis and Smith [33] aligns relatively closely to our own study by questioning whether occupational licensing of social services has improved quality or whether it simply represents rent-seeking behavior by incumbent workers. To do so, they exploited a federal staffing provision that required larger nursing homes to employ licensed social workers. They failed to find evidence that the increase in licensed staff improves patient care quality, patient quality of life, or the quality of social services offered by the nursing home [33].

## 3. Econometric Model and Data 

Our analysis incorporates a wide set of covariates that affect demand as well as the quality of nursing home services. These covariates represent four dimensions, including health, the economy, state policy, and socio-demographic factors. The basic equation is specified as:

(1) $$y_{i}=a+CON_{i}+s_{i}+h_{i}+c_{i}+p_{i}+\varepsilon_{i}$$

where *y_i_* is the service quality-related outcome variable for nursing home *i*. The key explanatory variable, *CON_i_*, is a binary variable that equals 1 if the state *i* operates nursing homes under CON regulations and 0 otherwise. Socio-demographic factors are represented by the vector *s*, health factors are included in the vector *h*, economic factors are represented by the vector *c*, while vector *p* represents the state policy variations. The error term is *ε_i_*.

### 3.1. Service Quality Variables

Previous research uses changes in the residents’ health, such as unplanned weight gains or losses, incontinence, and bedsores, as a measure of the quality of nursing home care. However, approximately 40–50% of all nursing home admissions are comprised of short stay, post-acute care residents, a feature that does not allow sufficient time for changes in the residents’ health to be observed [34]. Another limitation for reporting assessment is the lack of consumers’ utility for public reporting. Research suggests that condensing complex clinical information in a manner that is understandable to lay audiences is difficult [35].

As an alternative, the National Nursing Home Survey (NNHS) includes the total weighted health survey score, which is calculated by healthcare professionals, such as registered nurses, dieticians, and social workers. The survey examines the cleanliness of the facility, the appropriateness of staffing, and the satisfaction rate of a sample of the residents at every nursing home. The survey scores are shared with consumers as a tool to assist them in the evaluation of individual nursing homes. The methodology of the scoring requires meeting the requirements within a period of 9–15 months. The total weighted score is based on the date of the surveys, meaning that the most recent survey counts heavier than the older ones. In our analysis, the survey score for 2014 was multiplied by one, the score for 2013 was multiplied by two thirds, and the survey score for 2012 was multiplied by one third. The sum of these weighted survey scores was divided by three to generate the overall score of the nursing home facility.

The Centers for Medicare and Medicaid (CMM) use payrolls submitted by the participating nursing homes as a measure of service quality. In doing so, the CMM classify working hours into three types—reported working hours, expected working hours, and average working hours. Reported working hours (RWHs) are included on the submitted payrolls that are reported by the nursing homes. Expected working hours (EWHs) are based on the estimated average minutes for each nursing category produced by the CMM. The nursing categories used in the analysis were licensed practical nurse (LPN), certified nursing assistant (CNA), and registered nurse (RN). The basic rule for estimating the total expected working hours begins with the multiplication of minutes of each nurse type by the number of residents in the nursing facility, leading to the total of expected working hours for each nurse category. Lastly, average working hours were calculated as the mean of the reported working hours submitted to CMM for every state. These were used to compute adjusted working hours (AdjWH) per resident per day for each nursing category, such that:

(2) $$\mathrm{AdjWH}=\frac{\mathrm{RWH}}{\left(\mathrm{EWH}\times \mathrm{AWH}\times r\right)}$$

where the last factor, *r*, is the adjusting rate, which is incorporated in (2) so that the mean of AdjWH is equal to the mean of RWH. We included the ratings for both the staff and nurses, which captured the differences of the level of care by the residents’ level of severity. For instance, a nursing home that hosts residents with a higher level of chronic disease is expected to have more staffing and nurses in order to match the needs of those residents.

All certified nursing homes must meet more than 100 regulatory standards that are intended to protect residents. These supervisory standards represent the main guidelines for the inspection team to follow while examining many characteristics of the daily practice at the nursing homes, including the level of care given to residents, the procedures of care, the interactions between staff and residents, and the overall atmosphere of the nursing homes. Examples of these standards include appropriate management of medications, the security of residents from physical and mental abuse, and the nature of food storing and preparation. Conformity to these standards is determined by inspection teams, which are comprised of qualified personnel, including at least one registered nurse. These teams typically perform routine annual inspections, with additional inspections being performed depending on the outcomes of the annual investigations. The inspections consist of evaluations of the residents’ medical records, interviews with a sample of residents (including family members), and examinations of the qualifications of the nurses and the managerial staff. Nursing homes that provide services to residents that are funded by Medicare or Medicaid are obligated to make their most recent inspection scores accessible to the public, thus providing us with a third measure of nursing home service quality.

Finally, health complaints provide a fourth measurement of nursing home service quality. Each state’s health department includes a unit whose task is to identify nursing home complaints. In most cases, nurses can assist in the early detection of health complaints that negatively affect quality of life in a nursing home. These units typically receive complaints from a primary contact, process the intake of that complaint, and then proceed to an investigation that continues until the case is closed. This process emphasizes a mandate that long-term nursing care providers continuously meet Medicare, Medicaid, and state requirements. The definition of “improper care” typically involves complaints resulting from inaccurately or wrongfully prescribed medication and any other inappropriate treatments offered to nursing home residents. It also includes early discharging of a resident from a hospital to the nursing home or inadequate discharging procedures from the nursing home. A relatively large number of health claims are, of course, associated with a low quality of service. Our model uses the mean number of health deficiencies, the mean number of health complaints, and the natural log of the total dollar amount of fines resulting from noncompliance.

### 3.2. Covariates

Socio-demographic factors, *s_i_*, include the size of the 85+ years old population, population size, racial background, the female labor force participation rate, and a measure of each county’s disabled population. The size of the 85+ years old population relates positively to the demand for nursing home services, as this group often requires long-term care. The percentage of the population over 85 is calculated by dividing the number of the population over 85 by the total population for each county (multiplied by 100). The econometric model also controls for the size of the population by including the natural log of the population in each county. Variation across races is a source of bias as some minorities tend to serve their elderly at home due to socio-cultural considerations [36]. The model, therefore, includes a variable for racial background that divides the population into white, black, and others. Chiswick [5] finds a positive association between women participating in the labor force and the demand for nursing homes, given that unemployed women are most likely to provide in-home nursing care services to other family members. Therefore, the female labor force participation rate is included in the model. The percentage of the disabled population in each county is also included in the model, as states that have more disabled individuals face a higher demand for nursing home services [37].

Health factors included in the vector, *h_i_*, are nursing home occupancy rates, the percentage of individuals with health insurance, and Medicaid reimbursement rates. One of the most important covariates is the occupancy rate, which is a measure of the demand for nursing home services. The occupancy rate was calculated by dividing the number of residents in certified beds by the number of beds available in each nursing home. Health factors also include the percentage of individuals with health insurance. A larger health-insured population indicates a healthier population and an expected lower demand for nursing home services. However, healthy individuals are expected to live longer, and the need for a nursing home services will persist in the long run. State policy variations in Medicaid reimbursement rates are captured in *p_i_*. Reimbursement rates vary from one state to another. Higher Medicaid reimbursement rates may increase the supply of nursing home services by attracting new entrants or expanding the existing service providers [28]. Medicaid reimbursement rates may also be used by state legislatures to increase the number of bed vacancies.

Economic factors represented by the vector, *c*, include the natural log of mean retirement income, the unemployment rate, and the natural log of median property value for each county. The natural log of the mean retirement income is included in the model to account for the idea that individuals with higher retirement income are better equipped to secure the nursing home services of a private provider. Chiswick [5] suggests that patients with higher income are more likely to choose in-facility care, while Headen [38] fails to find evidence that the population with higher income seeks to have more convenient in-home service. Thus, it is possible that self-funded private patients replace Medicaid patients, which in turn may have a positive impact on service quality. Next, the rate of unemployment affects the demand for and the quality of the nursing home service. A higher unemployment rate reduces the ability to pay for nursing home services, while it increases the provision of within-family care as young unemployed adults tend to be available to provide the needed care to the elders of families. Lastly, the set of economic factors also includes the natural log of the median property value for each county. Higher property values, which indicate a higher level of income and a greater demand for a higher quality service, may influence the entry of new service providers or the expansion of existing nursing home facilities. On the other hand, higher property values are expected to deter new entrants and reduce competition, thus reducing the quality of nursing home care.

### 3.3. Data

This study uses a triangulation of cross-sectional data sources that cover the years 2012, 2013, and 2014. County-level demographic data from the 48 contiguous US states were extracted from the American Community Survey (ACS). This survey was conducted by the US Census Bureau and monitored by the US Federal Statistical System. Reimbursement rates data were collected from the Office of the Assistant Secretary for Planning and Evaluation, which is under the supervision of the US Department of Health and Human Services (DHHS). The DHHS determines the amount of funds available for state medical insurance using the Federal Medical Assistance Program (FMAP). The third source of nursing homes data is the Centers for Medicaid and Medicare Services. These data, which include more than 15,000 certified nursing homes nationwide, are available on the Nursing Home Compare website. This source provides all deficiencies reported by inspection teams as a result of noncompliance with state requirements. It also includes the cycle of the additional inspections, penalties, ownership types, counts of fines, the total dollar amount of fines, payment denials, providers’ characteristics (e.g., number of beds per nursing home and number of residents per nursing home), staff rating, and number of hours worked by staff, health, and fire safety. Political party affiliation also represents state-level data, and it is collected from the Pew Research Center (PRC).

The summary statistics for the service quality measures are reported in Table 1, and they are generally consistent with the idea that nursing home service quality tends to be lower in the CON states. For example, the weighted health survey scores are considerably lower in the CON states, as are the staffing hours for the various levels of nursing quality. On the other hand, the CON states produce marginally higher health inspection ratings and a smaller number of complaints. However, the total dollar amount of fines is substantially higher in the CON states than in the non-CON states, a comparison that reflects a lower level of compliance and service quality. Lastly, Table 1 also provides significance levels for the nonparametric Mann–Whitney U tests, which compare the distribution shape and central location of the outcome variables for the CON and non-CON states. These test the null hypothesis that the two samples come from the same population (i.e., that there is no statistically significant difference between the distributions of the two samples). As indicated in Table 1, the null hypothesis is rejected for 8 of the 10 service quality measures. 

Next, the summary statistics for the covariates are reported in Table 2, and they suggest that implementation of CON may be associated with a smaller number of nursing homes. Consequently, the patient-to-bed ratio is larger in CON states. Table 2 also indicates that CON states have a relatively higher occupancy rate (81% compared to 79% in non-CON states). The population of CON states is, on average, substantially smaller than that of the non-CON states. Nevertheless, there is a larger percentage of health-insured individuals in the CON states than in the non-CON states. The percentage of health-insured individuals is 3.5 percentage points higher in CON states. As also reported in Table 2, retirement and family incomes are slightly lower in the CON states, while property values in the CON states tend to be substantially lower than those in the non-CON states.

Table 2 shows that the older and disabled populations tend to concentrate more in the CON states than in non-CON states. The proportion of the disabled population, over the age of 65 and 85 years old, indicates higher percentages in the CON states by 1.9, 1.7, and 0.3 percentage points, respectively. A higher proportion of African Americans and a lower percentage of minorities live in the CON states, which conforms to the relatively lower income levels and higher rates of unemployment in the CON states. Lastly, Table 2 also provides significance levels for the nonparametric Mann–Whitney U tests, which compare the distribution shape and central location of the covariates for the CON and non-CON states. Again, these test the null hypothesis that the two samples come from the same population (i.e., that there is no statistically significant difference between the distributions of the two samples). As indicated in Table 1, the null hypothesis is rejected for 14 of the 15 covariates.

## 4. Estimation Results

The impact of CON regulations on the various iterations of for-profit nursing home service quality is shown in Table 3. First, CON states tend to produce significantly lower health survey scores than non-CON states. Using the mean scores for non-CON states, CON regulation appears to result in health survey scores that are about 11–16% lower. These results are robust across a variety of specifications. Next, the results in Table 3 also provide evidence that nursing homes in CON states replace labor hours provided by more-qualified licensed practical nurses (LPNs) with those provided by less-qualified certified nursing assistants (CNAs). According to our estimates, the typical for-profit nursing home in CON states substitutes about 6 min of work per resident by a CNA for about 2 min of work per resident by an LPN. Given that the typical nursing home has 90 residents, this means that nursing homes located in CON states reduce time worked by LPNs by 3 h.

The results in Table 3 do indicate that nursing home RNs and staff ratings are higher in CON states than they are in non-CON states. However, in the case of the former, the CON state premium is only about 5.5% (0.17 rating points), a result that falls to 0 when covariates for the state’s economy, health policies, and health factors are included in the model. Similarly, the CON state staff rating premium is only about 3.5% (0.10 rating points) when all covariates are included.

When health inspection ratings are used to measure nursing home service quality, nursing homes located in CON states outperform those in non-CON states. However, as in the case of nurse ratings, the CON state premium in this case is relatively small, ranging from only 2.9 to about 4%. Next, CON states also report fewer nursing home-related health complaints than their non-CON states counterparts. In this case, the regulatory advantage is a bit larger than before, ranging from about 4.8 to about 8.8%. The results in Table 3 generally hold when other types of nursing homes (i.e., not-for-profit and government) are included in the analysis. Even so, results related to the various health inspection ratings provided by state government health agencies that favor CON states are subject to interpretation according to the theory of economic regulation developed by Stigler [39] and Peltzman [40]. That is, if regulation is, according to the Stigler–Peltzman approach [39] (p. 3), “as a rule, acquired by the industry and designed and operated primarily for its benefit,” then it is not surprising that these particular service quality-related ratings are, as opposed to the health survey scores, higher under CON regulation.

In order to explore any bias from using mean statistics from skewed distributions, regressions employing median statistics for some of the outcomes variables were considered. These include both *Health Survey Scores* and *Fines*, given the relatively large standard errors reported in Table 1 for these two outcome variables. Regression results from four versions for each of these two outcome variables are reported in Table 4. As indicated there, CON states again tend to produce significantly lower health survey scores than non-CON states. In fact, using the median score for non-CON states, the parameter estimates attached to *Health Survey Scores* in Table 4 suggest that CON regulation appears to result in health survey scores that are about 21–25% lower. In terms of *Fines*, the results in Table 4 generally mirror those in Table 3.

The regression results presented in Table 5 pertain to other covariates that closely relate to quality of service in the nursing home industry. These are the reimbursement rate and the occupancy rate, both of which are expected to affect the operating cost of the nursing homes. In this case, our analysis covers both for-profit only and all types of nursing homes, including both not-for-profit and government nursing homes. This expansion in our analysis explores the possibility that these effects on nursing home quality differ by nursing home type. The results in Table 5 indicate that reimbursement rates are associated with a significantly higher quality of care, as measured by *Health Survey Scores*, in for-profit nursing homes. In this case, the return to service quality from the reimbursement rate is about 67% larger in non-CON states than it is where CON regulations prevail. However, when all nursing homes are included, as shown in the bottom portion of Table 5, the reimbursement rate return to *Health Survey Scores* is greater in CON states. Reimbursement rates are also positively associated with LPN ratings in CON states, regardless of nursing home type, while it is either unrelated to, or negatively related to, LPN ratings in non-CON states.

Expectedly, the occupancy rate is negatively associated with the quality of service in the nursing home industry. In all four cases, the reduction in *Health Survey Scores* associated with a one-point increase in occupancy rate is only about 0.5%. The relationship between occupancy rates and nurse ratings are also similar for for-profit and all nursing homes (including not-for-profit and government nursing homes), as well as across CON and non-CON states.

In order to explore any bias from using mean statistics from the skewed distribution of *Health Survey Scores*, regressions employing median statistics for this outcome variable are reported in Table 6. As indicated there, reimbursement rates are again associated with a significantly higher quality of care in for-profit nursing homes. However, in this case, the return to service quality from the reimbursement rate is larger in states where CON regulations prevail, without regard to nursing home type. On the other hand, while the occupancy rate is negatively associated with the quality of service in the nursing home industry, it is notably less so in non-CON states when all types of nursing homes are included.

We conducted a Hausman specification test for endogeneity [41], which showed that the previously discussed CON estimates are unbiased and that the CON variable can be treated as exogenous. However, there is a concern that a state’s decision regarding the implementation of CON regulations is endogenous to the quality of nursing home care. Thus, two-stage least squares (2SLS) estimates were obtained in order to check the robustness of the results. In this case, Republican Party affiliation is used as an instrumental variable (IV) for CON states. Party affiliation data by state (for 2014) were collected from the Pew Research Center. We find that an increase of one percentage point in Republican Party affiliation of the state lead to a 0.5 percentage point increase in the likelihood of observing CON regulations (*p*-value < 0.05). Moreover, the instrument is uncorrelated with the residual of the main equation (*p*-value > 0.10), which indicates exogeneity to the outcome variables. These results are indicative of a strong instrumental variable that has desirable asymptotic properties. Results from an IV approach for both for-profit nursing homes and for all nursing homes are presented in Table 7.

As indicated in Table 7, CON states tend to produce significantly lower health survey scores than non-CON states. Using the mean scores for non-CON states, CON regulation appeares to result in health survey scores that are about 24% (19.7 out of 81.4 survey points) lower in the case of for-profit nursing homes. Additionally, CON regulation tends to reduce the work hours of both RNs and LPNs, which, in the latter case, is statistically significant. In that case, CON appears to reduce the employment of LPN by 0.09 h (i.e., about 5.3 min) per resident per day. At the same time, CON regulation is associated with an increase in hours worked by CNAs—by 0.3 h (i.e., about 20 min) per resident per day—a result that suggests a substitution of CNA labor for LPN labor. However, in this case, each unit of LPN labor is replaced by 3.7 times that in units of CNA labor, whereas the earlier ratio is smaller, at three times that in units of CNA labor (i.e., 6 min of CNA time for 2 min of LPN time).

Next, CON regulation is associated with higher staff and inspection ratings, a result that is subject to interpretation according to the theory of economic regulation developed by Stigler [39] and Peltzman [40], in which case they are unsurprising. Lastly, CON regulation fails to produce fewer health complaints using the 2SLS approach. Thus, overall, the results from the IV approach presented in Table 7 are even less favorable to CON regulation of nursing homes than the original results shown in Table 3.

## 5. Conclusions

This study employs a variety of outcome variables to investigate the relationship between quality of the service and the presence of certificate-of-need regulation in the nursing home industry. To do so, it makes use of a panel of county-level demographic data from the 48 contiguous US states that covers the 3-year period from 2012 through 2014. Instrumental variables results indicate that health survey scores that are tabulated by healthcare professionals, such as registered nurses, dieticians, and social workers, are about 18–24% lower for nursing homes located in states with CON regulation, depending on the type of nursing home (e.g., for-profit, not-for-profit, etc.).

Additionally, CON regulation tends to reduce the employment of both registered nurses and licensed practical nurses, two sources of relatively high-quality nursing care, while at the same time increasing the employment of certified nursing assistants, a lower-quality nursing care option. When healthcare services provided by registered nurses are made available to nursing home residents, they are, according to our results, rated lower in CON states than in non-CON states. Lastly, the presence of CON regulation fails to result in fewer health complaints in the nursing home industry.

One limitation of the current study is the treatment of economic regulation in a binary manner. Future research could extend the literature by combining certificate-of-need regulation with other forms of state regulation to create a Likert-type index that could be used in regression analysis in order to better parse the impact of economic regulation on service quality in the nursing home industry. Additionally, future research could approach the subject in a manner similar to Upadhyaya, Raymond, and Mixon’s [42] replication of the classic study on the regulation of electric utilities by Stigler and Friedland [43]. This approach would combine an analysis of the impact of regulation on service quality in the nursing home industry with an exploration into how service quality in that industry impacts the likelihood of observing certificate-of-need (or other) regulation of nursing homes in a state.

## Figures and Tables

**Table 1:** Summary statistics for service quality variables.

Service Quality Variables	CON States	Non-CON States	Mann-Whitney U Test
*Health Survey Scores*	51.61 (65.09)	81.35 (84.26)	***
*Registered Nurse (RN)*	0.53 (0.23)	0.53 (0.26)	***
*Certified Nursing Assistant (CNA)*	2.39 (0.56)	2.42 (0.63)	
*Licensed Practical Nurse (LPN)*	1.06 (0.41)	1.08 (0.45)	*
*Nurse*	3.84 (0.74)	3.88 (0.83)	
*RN Rating*	3.26 (1.18)	3.21 (2.27)	***
*Staff Rating*	3.04 (1.09)	3.02 (1.19)	*
*Health Inspection Rating*	2.72 (1.28)	2.63 (1.27)	*
*Health Complaints*	1.81 (2.49)	2.43 (3.29)	**
*Fines*	10,281 (47,892)	6702 (26,506)	***
*Positive Fines*	38,674 (82,733)	26,159 (47,617)	
Median of *Health Survey Scores*	31.33	49.67	
Median of *Positive Fines*	10,433	8651	
Average Number of Nursing Homes per County	8.61	7.56	
Number of Nursing Homes	9295	5320	
Number of States	34	14	

Notes: The numbers in parentheses are standard errors. Data were reported for each nursing home, after which the averages were calculated for certificate-of-need (CON) and non-CON states. *RN* is adjusted RN staffing hours per resident per day. *CNA* is adjusted CNA staffing per resident per day. *LPN* is adjusted LPN staffing hours per resident per day. *Nurse* is adjusted total nurse staffing hours per resident per day. ***(**)[*] indicates the 0.001(0.01)[0.05] level of significance for the Mann–Whitney U test.

**Table 2:** Summary Statistics for the Covariates.

Covariates	CON States	Non-CON States	Mann–Whitney U Test
*Occupancy Rates*	81% (17.27)	79% (18.68)	***
*Population*	485,000 (836,335)	1,740,358 (2,290,548)	***
*Retirement Income*	22,054 (4559)	23,490 (4478)	***
*Family Income*	79,100 (21,589)	83,840 (18,639)	***
*Health Insured*	87.0% (4.66)	83.6% (5.80)	**
*Unemployment Rate*	6.0% (1.49)	5.7% (1.57)	***
*Disability*	13.6% (4.77)	11.7% (3.84)	***
*Property Value*	170,500 (96,157)	223,677 (148,647)	***
*Reimbursement Rate*	60% (7.38)	57% (6.04)	*
*Population over 65*	15.0% (5.17)	13.3% (4.86)	**
*Population over 85*	2.0% (0.34)	1.7% (0.38)	***
*White*	75.7% (8.88)	75.0% (9.60)	***
*Black*	15.0% (8.66)	7.7% (3.25)	***
*Minority*	9.3% (5.05)	17.5% (10.15)	***
*Female LFPR*	58.5% (3.05)	58.7% (2.95)	
Number of States	34	14	

Notes: Data were reported for each nursing home, after which the averages were calculated for CON and non-CON states. The numbers in parentheses are standard errors. ***(**)[*] indicates the 0.001(0.01)[0.05] level of significance for the Mann–Whitney U test.

**Table 3:** The impact of CON on service quality in for-profit nursing homes.

Dependent Variables	Coefficient Estimates for CON				Number of Observations
	(1)	(2)	(3)	(4)	
*Health Survey Scores*	−13.72 *** (2.247)	−9.469 *** (2.269)	−9.849 *** (2.274)	−9.935 *** (2.281)	8322
*RN*	0.025 (0.073)	0.002 (0.073)	−0.001 (0.073)	−0.008 (0.073)	8255
*CNA*	0.105 *** (0.018)	0.096 *** (0.018)	0.089 *** (0.018)	0.103 *** (0.018)	8255
*LPN*	−0.038 *** (0.013)	−0.025 ** (0.013)	−0.023 * (0.013)	−0.033 ** (0.013)	8255
*RN Rating*	0.175 *** (0.013)	0.043 (0.036)	0.025 (0.036)	−0.012 (0.036)	8255
*Staff Rating*	0.248 *** (0.034)	0.146 *** (0.035)	0.126 *** (0.034)	0.107 *** (0.035)	8255
*Health Inspection Rating*	0.105 *** (0.040)	0.075 * (0.041)	0.080 * (0.041)	0.094 ** (0.041)	8322
*Health Complaints*	−0.213 ** (0.087)	−0.116 (0.088)	−0.146 * (0.088)	−0.180 ** (0.089)	8244
*Fines*	0.052 (0.097)	0.103 (0.100)	0.081 (0.100)	0.019 (0.101)	2073
Variables Included:					
*Socio-Demographics*	yes	yes	yes	yes	
*Economic*	no	yes	yes	yes	
*State Policies*	no	no	yes	yes	
*Health Factors*	no	no	no	yes	

Notes: Ordinary least squares (OLS) was used to estimate the reported coefficients. The first model in the table includes the socio-demographics covariates only, while the fourth model includes all of the covariates. The numbers in parentheses are standard errors. ***(**)[*] indicates the 0.001(0.01)[0.05] level of significance.

**Table 4:** The impact of CON on service quality in for-profit nursing homes.

Dependent Variables	Coefficient Estimates for CON				Number of Observations
	(1)	(2)	(3)	(4)	
*Health Survey Scores*	−12.65 *** (1.371)	−10.35 *** (1.421)	−10.24 *** (1.409)	−10.71 *** (1.367)	8322
*Fines*	0.018 (0.155)	0.026 (0.161)	−0.007 (0.157)	−0.099 (0.152)	2073
Variables Included:					
*Socio-Demographics*	yes	yes	yes	yes	
*Economic*	no	yes	yes	yes	
*State Policies*	no	no	yes	yes	
*Health Factors*	no	no	no	yes	

Notes: Ordinary least squares (OLS) was used to estimate the reported coefficients. The first model in the table includes the socio-demographics covariates only, while the fourth model includes all of the covariates. The numbers in parentheses are standard errors. *** indicates the 0.001 level of significance.

**Table 5:** The impact of other covariates on service quality by nursing home type.

	For-Profit Nursing Homes					
	CON States			Non-CON States		
Covariates	*Health Survey Scores*	*CNA*	*LPN*	*Health Survey Scores*	*CNA*	*LPN*
*Reimbursement Rate*	0.971 *** (0.248)	−0.001 (0.002)	0.012 *** (0.002)	1.636 *** (0.595)	0.001 (0.003)	−0.004 (0.004)
*Occupancy Rate*	−0.402 *** (0.054)	−0.002 *** (0.000)	−0.002 *** (0.000)	−0.421 *** (0.101)	−0.004 *** (0.001)	−0.001 (0.001)
*n*	5621	5567	5567	2701	2658	2658
	**All Nursing Homes**					
	**CON States**			**Non-CON States**		
	***Health Survey Scores***	***CNA***	***LPN***	***Health Survey Scores***	***CNA***	***LPN***
*Reimbursement Rate*	1.000 *** (0.183)	0.001 (0.002)	0.010 *** (0.002)	0.564 (0.354)	−0.002 (0.002)	−0.006 * (0.003)
*Occupancy Rate*	−0.389 *** (0.041)	−0.003 *** (0.000)	−0.003 *** (0.001)	−0.369 *** (0.064)	−0.003 *** (0.000)	0.002 *** (0.001)
*n*	9219	9059	9059	5267	5165	5165

Notes: The upper half of the table pertains to for-profit nursing homes, while the lower half includes all nursing homes. The right side of the table displays the effect on nursing homes in CON states, while the left side of the table displays the effect on nursing homes in the non-CON states. The numbers in parentheses are standard errors. ***[*] indicates the 0.001[0.05] level of significance.

**Table 6:** The impact of other covariates on service quality by nursing home type.

	For-Profit Nursing Homes	
	CON States	Non-CON States
	*Health Survey Scores*	*Health Survey Scores*
*Reimbursement Rate*	0.261 * (0.143)	0.106 * (0.052)
*Occupancy Rate*	−0.297 *** (0.031)	−0.291 *** (0.064)
*n*	5621	2701
	**All Nursing Homes**	
	**CON States**	**Non-CON States**
	***Health Survey Scores***	***Health Survey Scores***
*Reimbursement Rate*	0.191 * (0.105)	−0.006 (0.209)
*Occupancy Rate*	−0.266 *** (0.024)	−0.180 *** (0.038)
*n*	9219	5267

Notes: The upper half of the table pertains to for-profit nursing homes, while the lower half includes all nursing homes. The right side of the table displays the effect on nursing homes in CON states, while the left side of the table displays the effect on nursing homes in the non-CON states. The numbers in parentheses are standard errors. ***[*] indicates the 0.001[0.05] level of significance.

**Table 7:** The impact of CON on service quality in for-profit nursing homes, instrumental variable (IV) estimates.

	Coefficient Estimates for CON	
Dependent Variables	For-Profit Nursing Homes	*n*
*Health Survey Scores*	−19.70 *** (4.125)	8322
*RN*	−0.010 (0.013)	8255
*CNA*	0.312 *** (0.033)	8255
*LPN*	−0.088 *** (0.021)	8255
*RN Rating*	0.155 (0.129)	8255
*Staff Rating*	0.207 ** (0.100)	8255
*Health Inspection Rating*	0.488 *** (0.120)	8322
*Health Complaints*	0.373 (0.254)	8244
*Fines*	0.060 (0.374)	2073

Notes: The numbers in parentheses are standard errors. ***(**) indicates the 0.001(0.01) level of significance.
